# Hashimoto’s Thyroiditis and Female Fertility: Endocrine, Immune, and Microbiota Perspectives in Assisted Reproduction—A Narrative Review

**DOI:** 10.3390/biomedicines13061495

**Published:** 2025-06-18

**Authors:** Emilia Cristina Popa, Laura Maghiar, Teodor Andrei Maghiar, Ilarie Brihan, Laura Monica Georgescu, Bianca Anamaria Toderaș, Liliana Sachelarie, Anca Huniadi

**Affiliations:** 1 Department of Medical Sciences, Faculty of Medicine and Pharmacy, University of Oradea, 1st December Square 10, 410073 Oradea, Romania; emilia.cristinapopa@gmail.com (E.C.P.); amalfilaura27@yahoo.com (L.M.G.); bianca.toderas96@gmail.com (B.A.T.); 2 Department of Psychoneurosciences and Recovery, Faculty of Medicine and Pharmacy, University of Oradea, 1st December Square 10, 410073 Oradea, Romania; laura.maghiar@uoradea.ro (L.M.); brihan_drm@yahoo.com (I.B.); 3 Department of Surgery, Faculty of Medicine and Pharmacy, University of Oradea, 1st December Square 10, 410073 Oradea, Romania; teodormaghiar@yahoo.com (T.A.M.); ancahuiadi@gmail.com (A.H.); 4 Preclinical Sciences Department, Faculty of Medicine, Apollonia University, 700511 Iasi, Romania

**Keywords:** thyroid autoantibodies, ovarian reserve, immune dysregulation

## Abstract

Hashimoto’s thyroiditis is the most prevalent autoimmune thyroid disorder, and it disproportionately affects women of reproductive age. Its impact on fertility and assisted reproductive technologies [ART] has become an area of growing clinical interest. Thyroid autoimmunity can influence female reproductive health through multiple interconnected mechanisms, including subtle thyroid hormone imbalances, reduced ovarian reserve, altered endometrial receptivity, and dysregulated immune responses. Subclinical hypothyroidism and the presence of anti-thyroid antibodies have been linked to increased miscarriage risk and reduced success rates in ART, particularly in intracytoplasmic sperm injection (ICSI) cycles. Although levothyroxine supplementation is widely used, its benefits in euthyroid women remain uncertain. Recent studies suggest that gut microbiota may modulate immune function and affect fertility outcomes among women with autoimmune thyroid conditions. This narrative review synthesizes findings from a broad literature base of over 40 peer-reviewed publications published between 2010 and 2025, with 30 of the most relevant and methodologically robust studies selected for detailed analysis. The review integrates clinical, endocrine, immunological, and microbiome-related perspectives. The evidence supports the need for personalized fertility management in women with Hashimoto’s thyroiditis and highlights directions for future research into immune and microbiota-targeted therapies.

## 1. Introduction

This review aims to comprehensively map and synthesize current evidence on the role of Hashimoto’s thyroiditis in female infertility and ART success, integrating hormonal, immunological, and microbiota-related mechanisms while identifying key research gaps and future directions. The prevalence of Hashimoto’s autoimmune thyroiditis is increasing, particularly affecting young women. Given the essential role of thyroid hormones in regulating the menstrual cycle, ovulation, and pregnancy maintenance, this condition becomes a crucial factor in evaluating infertility and the success of assisted reproductive treatments [1,2].

The inclusion criteria for this narrative review encompassed peer-reviewed articles published between 2010 and 2025, written in English, which examined the relationship between Hashimoto’s thyroiditis and female fertility. Eligible studies addressed at least one of the following: hormonal and immunological factors affecting fertility, assisted reproductive outcomes (e.g., IVF/ICSI success), or the role of the gut microbiota in thyroid autoimmunity. Studies were selected based on methodological quality and relevance to the review’s objectives.

This article stands out through its interdisciplinary approach, integrating insights from endocrinology, immunology, reproductive medicine, and microbiology. The review comprehensively explains how Hashimoto’s thyroiditis impacts female fertility by synthesizing knowledge from diverse scientific domains. The review is based on a rigorous selection of studies published between 2010 and 2025, reflecting a growing and up-to-date body of clinical evidence. The inclusion of emerging concepts—such as the role of gut microbiota in modulating immune function—underscores the evolving nature of this field. Recent epidemiological data indicate that thyroid autoimmune disorders tend to be more prevalent in populations with lower socioeconomic status and are influenced by both genetic (e.g., HLA, CTLA4, PTPN22, IL2R, CD14, CD40) and environmental factors such as smoking, stress, viral/bacterial infections, and exposure to certain chemicals [3]. The role of thyroid hormones in female fertility is critical. These hormones regulate menstrual regularity, ovulatory function, implantation, and fetal development [3,4]. Disruptions in thyroid function, even at a subclinical level, can lead to menstrual irregularities and ovulatory dysfunction, which are common causes of infertility [5].

Thyroid dysfunction, particularly autoimmune thyroiditis, has been associated with a 2- to 4-fold increased risk of miscarriage and preterm delivery in pregnant women positive for anti-thyroid antibodies [6]. Given these findings, managing thyroid dysfunction is essential not only for maternal health but also for improving reproductive outcomes. With the increasing reliance on assisted reproductive techniques (ART), including in vitro fertilization (ETA) and intracytoplasmic sperm injection (ICSI), there is growing interest in understanding how thyroid autoimmunity affects treatment success. Numerous studies have explored the interplay between thyroid autoimmunity and ART outcomes, leading to ongoing debates regarding the management of euthyroid women with positive thyroid autoantibodies [7,8].

This review aims to present the current evidence regarding the impact of Hashimoto’s thyroiditis on female fertility and ART success, outlining pathophysiological mechanisms, clinical implications, and therapeutic strategies based on recent research and international guidelines.

By bridging evidence across disciplines, the review serves as both a scientific synthesis and a practical guide for clinicians and researchers alike.

## 2. Pathophysiological Mechanisms

**Figure 1:**
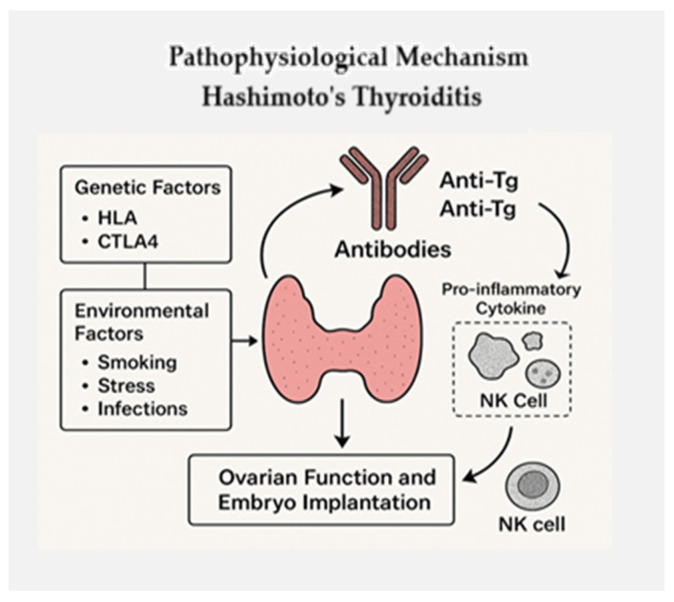
Pathophysiological mechanisms of Hashimoto’s thyroiditis. Pathophysiological mechanisms of Hashimoto’s thyroiditis. The authors created the original figure with AI assistance (ChatGPT v4, OpenAI).

Figure 1 highlights the intricate interplay of genetic, environmental, and immunological factors involved in the onset of Hashimoto’s thyroiditis and its reproductive consequences.

The pathogenesis of Hashimoto’s thyroiditis involves a complex interplay between innate and adaptive immune responses. At the cellular level, the loss of self-tolerance is primarily driven by autoreactive CD4+ T helper cells, particularly Th1 and Th17 subtypes, which infiltrate thyroid tissue and release pro-inflammatory cytokines such as IFN-γ, IL-17, and TNF-α [4,9,10]. These cytokines contribute to the activation of cytotoxic CD8+ T cells and local macrophages, leading to progressive thyrocyte destruction. Concurrently, a deficiency in regulatory T cells (Tregs), which normally suppress autoreactive lymphocytes, exacerbates immune dysregulation [9]. On the molecular level, polymorphisms in genes such as CTLA4, CD40, and PTPN22 have been shown to affect co-stimulatory signaling and antigen presentation, promoting a heightened autoimmune state [5,11]. Additionally, aberrant activation of the JAK/STAT signaling pathway in immune cells enhances transcription of pro-inflammatory mediators, sustaining the autoimmune milieu [9,12]. These systemic immune alterations may extend beyond the thyroid, disrupting ovarian folliculogenesis, impairing oocyte maturation, and reducing endometrial receptivity, thereby contributing to infertility in women with Hashimoto’s thyroiditis [6,13,14].

In addition to cellular immune pathways, several other pathophysiological mechanisms have been implicated in the link between Hashimoto’s thyroiditis and infertility. Specific autoantibodies such as anti-TPO and anti-thyroglobulin may cross the follicular barrier and negatively impact the ovarian microenvironment, affecting oocyte maturation and embryo quality [6,13]. Moreover, women with HT often exhibit increased systemic oxidative stress, which impairs mitochondrial function in oocytes and compromises endometrial receptivity, thereby interfering with successful implantation [9,11]. Simultaneously, gut microbiota dysbiosis contributes to excessive polarization of Th17 responses and sustains systemic inflammation, exacerbating the autoimmune burden on the reproductive axis [12,14,15,16,17,18].

Genetic predispositions—such as polymorphisms in HLA and CTLA4—interact with environmental triggers like smoking, stress, and infections to initiate an autoimmune response. This immune activation produces anti-thyroid antibodies, mainly anti-thyroglobulin (Anti-Tg) and anti-thyroid peroxidase (ATPO), specifically targeting thyroid tissue. Alongside these antibodies, elevated levels of pro-inflammatory cytokines, such as interleukin-17α (IL-17α), and the activation of natural killer (NK) cells contribute to ongoing immune-mediated damage. The resulting thyroid inflammation and tissue destruction can disrupt hormonal balance and negatively impact ovarian function and embryo implantation, even in women with normal thyroid hormone levels. This figure underscores how immune dysregulation in Hashimoto’s extends beyond thyroid dysfunction, affecting female reproductive health through hormonal and immunological pathways.

Hashimoto’s thyroiditis is immune-mediated, characterized by the production of anti-thyroid peroxidase (ATPO) and anti-thyroglobulin (A-Tg) antibodies, which may influence ovarian function and embryo implantation. Genetic and environmental factors contribute to disease onset. Increased NK cell activity and the presence of pro-inflammatory cytokines such as IL-17α can negatively affect oocytes and ovarian reserve.

Among the key immunological factors involved in Hashimoto’s thyroiditis, anti-thyroid peroxidase (Anti-TPO) and anti-thyroglobulin (Anti-Tg) antibodies play a central role, being closely associated with ovarian inflammation and an increased risk of miscarriage. Additionally, the pro-inflammatory cytokine interleukin-17α (IL-17α) has been implicated in the reduction in oocyte quality and impaired implantation potential. Natural killer (NK) cells, which are often hyperactivated in autoimmune conditions, contribute to reduced endometrial receptivity and may interfere with embryo implantation. Moreover, KIR-HLA-C mismatches, representing an immunogenetic incompatibility between maternal killer cell receptors and fetal HLA ligands, have been linked to lower implantation success rates in assisted reproductive technology (ART) cycles, Table 1.

The etiology involves a complex interplay between genetic predisposition and environmental triggers. Key genetic factors include polymorphisms in immune-regulatory genes such as HLA, CTLA4, PTPN22, IL2R, CD14, and CD40, which are involved in antigen presentation, T-cell regulation, and cytokine signaling [3]. Environmental contributors like smoking, chronic psychological stress, viral or bacterial infections, and exposure to toxic agents are also strongly implicated in the disease’s onset and progression [3,4]. Women with Hashimoto’s thyroiditis are more likely to develop concurrent autoimmune ovarian dysfunction, characterized by reduced ovarian reserve and disrupted folliculogenesis. One hypothesized mechanism is the immune-mediated alteration of the ovarian microenvironment, marked by elevated levels of pro-inflammatory cytokines, particularly interleukin-17α [IL-17α], which can impair oocyte quality and function [6]. Additionally, aberrant activation of natural killer [NK] cells and their receptor-ligand dynamics, such as killer immunoglobulin-like receptors [KIRs] interacting with HLA-C antigens, play a significant role in the pathogenesis of Hashimoto’s thyroiditis. These interactions may influence thyroid autoimmunity, implantation success, and placental development [4].

This immune dysregulation can result in fluctuating thyroid hormone levels, contributing to menstrual irregularities and ovulatory dysfunction, both critical factors in female fertility. As such, thyroid autoimmunity represents both a systemic and reproductive immunological challenge that warrants close clinical attention in women seeking to conceive [5,6].

The described immunological disturbances in Hashimoto’s thyroiditis particularly Th1/Th17 polarization, impaired Treg function, and elevated pro-inflammatory cytokines are associated with clinically relevant outcomes in ART. These include reduced oocyte maturation, poorer embryo quality, and lower implantation and live birth rates [6,13,14]. Moreover, elevated anti-thyroid antibodies have been linked to increased miscarriage risk even in euthyroid women undergoing IVF, further highlighting the clinical relevance of immune dysregulation in thyroid autoimmunity [11,12].

## 3. Ovarian Reserve and Thyroid Dysfunction

Ovarian reserve refers to the quantity and quality of a woman’s remaining oocytes, which naturally decline with age. However, in women with autoimmune conditions such as Hashimoto’s thyroiditis, this decline may be accelerated by chronic immune activation and inflammatory responses. The ovarian reserve is typically assessed using markers such as anti-Müllerian hormone (AMH), follicle-stimulating hormone (FSH), estradiol (E2), and antral follicle count (AFC) by transvaginal ultrasound.

Hashimoto’s thyroiditis is frequently associated with subclinical hypothyroidism, defined by elevated thyroid-stimulating hormone (TSH) levels with normal free thyroxine (FT4). Although often asymptomatic, this condition can subtly affect the hypothalamic-pituitary-ovarian axis, impair folliculogenesis, and reduce AMH levels, suggesting diminished ovarian reserve even in women of reproductive age. Additionally, elevated FSH and decreased AFC have been reported in patients with thyroid autoimmunity, indicating compromised ovarian responsiveness.

The relationship between thyroid function and ovarian reserve is complex and possibly bidirectional. Thyroid hormones influence granulosa cell function and follicular maturation, while chronic low-grade inflammation seen in autoimmune thyroiditis may disrupt the ovarian microenvironment. Studies suggest that subclinical hypothyroidism and the presence of thyroid autoantibodies are associated with lower AMH concentrations, although findings across studies remain inconsistent.

In the context of Hashimoto’s thyroiditis, subclinical hypothyroidism is generally defined as elevated serum TSH levels (commonly > 4.0–4.5 mIU/L) with normal free thyroxine (FT4) concentrations. However, this definition varies across studies, particularly those involving assisted reproductive technology (ART), where stricter thresholds such as TSH > 2.5 mIU/L are often applied to optimize implantation outcomes [13,14,24]. This lack of consensus may partly explain the conflicting findings regarding the impact of thyroid autoimmunity on ovarian reserve and ART success rates.

In clinical practice, assessing ovarian reserve in women with Hashimoto’s thyroiditis is essential, particularly when planning assisted reproductive treatments. Monitoring AMH and FSH in conjunction with TSH can help guide ovarian stimulation protocols. Furthermore, recent research has shown that levothyroxine therapy in subclinically hypothyroid patients may improve ovarian parameters, although further large-scale studies are warranted.

Overall, a comprehensive evaluation of both thyroid and reproductive endocrine function is recommended for optimizing fertility outcomes in women with autoimmune thyroid disease, Table 2.

Recent evidence indicates that thyroid hormone replacement therapy with levothyroxine may improve ovarian reserve in some women with Hashimoto’s thyroiditis, as reflected by rising AMH levels post-treatment [6,7]. However, these findings remain under investigation, and further randomized controlled trials are needed to establish definitive clinical guidelines.

Given the interconnection between thyroid function and reproductive hormones, careful screening and management of thyroid status, even at subclinical stages, is essential for optimizing fertility outcomes in women seeking pregnancy.

While the standard of care for overt hypothyroidism remains levothyroxine (LT4) replacement, its role in euthyroid women with Hashimoto’s thyroiditis undergoing assisted reproduction remains controversial. Recent studies indicate that LT4 supplementation may improve pregnancy outcomes, particularly in women with positive TPO antibodies, by stabilizing thyroid function during ovarian stimulation and early gestation [5,13]. However, other trials report limited or no benefit, especially in euthyroid patients with low-normal TSH levels, underscoring the need for individualized treatment strategies and further randomized controlled trials [14,29].

## 4. Clinical Implications in Assisted Reproduction

Numerous studies show a correlation between the presence of thyroid autoantibodies and higher rates of miscarriage or implantation failure. In euthyroid women, levothyroxine supplementation did not significantly reduce subfertility rates. However, some data suggest improved implantation rates in patients with elevated ATPO. In ICSI cycles, patients with thyroid autoimmunity showed poorer reproductive outcomes compared to those undergoing IVF, Table 3. Subclinical hypothyroidism, prevalent among women with Hashimoto’s thyroiditis, may go unnoticed due to the absence of overt symptoms; yet, it still negatively affects fertility and pregnancy outcomes [7,8]. Although thyroid hormone replacement with levothyroxine has shown benefits in women with overt hypothyroidism, its role in euthyroid women with thyroid autoimmunity remains debated. A 2024 study conducted by the University of Rochester concluded that levothyroxine supplementation in euthyroid women with positive thyroid antibodies did not reduce subfertility or miscarriage rates [20].

Nonetheless, other studies have indicated that levothyroxine treatment in women with subclinical hypothyroidism and elevated ATPO levels may enhance implantation and conception rates, highlighting the potential benefit of a tailored therapeutic approach [8].

In terms of assisted reproductive techniques, particularly intracytoplasmic sperm injection [ICSI], research shows that patients with autoimmune thyroiditis may experience lower fertilization rates, reduced clinical pregnancy rates, and an increased risk of early miscarriage compared to those undergoing conventional in vitro fertilization (ETA) [31,32]. A meta-analysis of randomized controlled trials found that while levothyroxine did not significantly impact clinical pregnancy or live birth rates, it was associated with a decreased rate of miscarriage among women with thyroid autoimmunity [33].

These findings suggest that while thyroid autoimmunity does not preclude successful ART outcomes, it may influence reproductive prognosis and should be considered during fertility assessments. Personalized management strategies, including close endocrine monitoring and selective use of thyroid hormone therapy, may help optimize outcomes for these patients.

## 5. International Guidelines and Recommendations

The American Society for Reproductive Medicine recommends maintaining TSH levels below 2.5 mIU/L before initiating IVF. European guidelines (ETA)suggest using ICSI in infertile women with thyroid autoimmunity, though evidence for its efficacy is mixed. Levothyroxine supplementation is recommended for patients with elevated TSH, but not for euthyroid individuals.

Similarly, the European Thyroid Association (ETA)advises that achieving a TSH concentration below this threshold may improve reproductive outcomes in women with autoimmune thyroid disease, especially during ovarian hyperstimulation, when estrogen levels elevate thyroid-binding globulin and alter free thyroid hormone levels [13].

Although both ASRM and ETA acknowledge the increased prevalence of thyroid autoantibodies among infertile women, particularly those with polycystic ovary syndrome (PCOS) or idiopathic infertility, they differ slightly in their therapeutic approaches. The ETA has suggested the use of intracytoplasmic sperm injection [ICSI] over conventional IVF in women with thyroid autoimmunity to potentially bypass immunological barriers at the zona pellucida level. However, this recommendation remains controversial, as recent large-scale cohort studies have not consistently demonstrated improved outcomes with ICSI in this subgroup [11].

Levothyroxine supplementation is widely accepted for patients with overt or subclinical hypothyroidism, especially those with TSH levels above the upper limit of the normal range. However, the role of levothyroxine in euthyroid women with positive thyroid antibodies remains debated. While some data suggest that supplementation may reduce miscarriage risk [10], randomized trials have failed to show a consistent benefit in terms of increasing live birth or pregnancy rates [20,30].

Although levothyroxine remains the first-line treatment for Hashimoto’s thyroiditis, some studies have also explored the adjunctive use of low-dose glucocorticoids—such as prednisone—in selected cases to modulate systemic inflammation and autoimmunity, especially in patients with coexisting autoimmune conditions. However, these approaches are not yet supported by standard fertility guidelines and should be considered investigational [11].

Given the heterogeneity in clinical trial results, current guidelines differ in their recommendations for levothyroxine supplementation in euthyroid women with thyroid autoimmunity. While some advocate for routine use to reduce miscarriage risk, others call for individualized assessment. This divergence underscores the need for further evidence to establish clear, universally accepted protocols [12,30,33].

In light of this evidence, major clinical guidelines advocate for individualized management strategies. These should consider both TSH levels and the presence of thyroid autoantibodies when planning fertility treatments, rather than applying a one-size-fits-all therapeutic protocol.

## 6. Gut Microbiota and Research Directions

Microbiota imbalances may influence autoimmunity and reproductive prognosis. A potential link between intestinal dysbiosis and the production of autoantibodies is suggested, but further studies are needed to elucidate the mechanisms.

Emerging research underscores the potential role of gut microbiota in modulating immune responses and influencing the course of autoimmune diseases, including Hashimoto’s thyroiditis. The gastrointestinal tract houses trillions of microorganisms that contribute to immune system regulation by producing metabolites and short-chain fatty acids and interacting with host immune cells [13].

**Figure 2:**
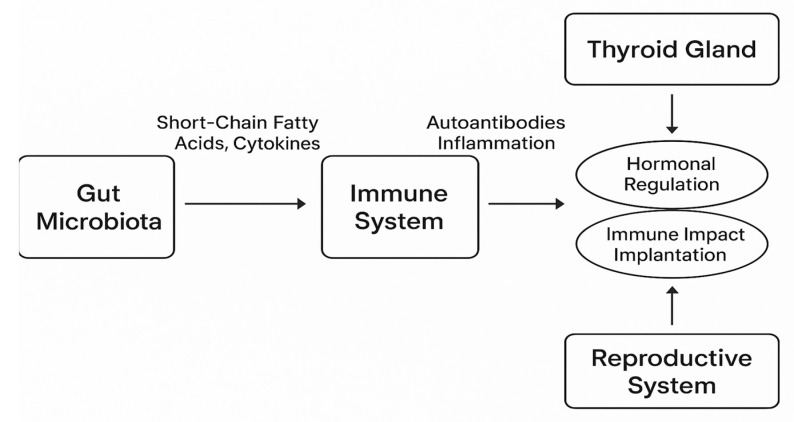
The gut–thyroid–reproduction axis in Hashimoto’s thyroiditis.

Figure 2 illustrates the bidirectional interactions between the gut microbiota, immune system, thyroid gland, and female reproductive system. Gut microbial metabolites such as short-chain fatty acids (SCFAs) and microbial byproducts influence immune activity by modulating cytokine production and systemic inflammation. This, in turn, affects thyroid autoimmunity through mechanisms such as increased production of anti-thyroid antibodies (e.g., anti-TPO, anti-Tg) and immune dysregulation. The thyroid gland, affected by this autoimmune response, may alter hormonal outputs (e.g., TSH, FT4), which are essential for ovarian function, endometrial receptivity, and embryo implantation. Additionally, inflammatory mediators and immune imbalances can directly impair reproductive processes, potentially reducing fertility and ART success. This axis underscores the multifactorial nature of infertility in women with Hashimoto’s thyroiditis and suggests emerging targets for therapeutic intervention, including microbiota modulation.

In patients with autoimmune thyroid disease, particularly Hashimoto’s thyroiditis, disruptions in gut microbial diversity also known as intestinal dysbiosis have been linked to increased systemic inflammation and aberrant immune activation. These imbalances may foster the development of thyroid autoantibodies by impairing immune tolerance mechanisms [12,32,34,35,36].

There is growing interest in the hypothesis that gut microbiota may indirectly influence reproductive health by contributing to systemic immune dysregulation. This could potentially impair ovarian function, endometrial receptivity, or the immune tolerance required for embryo implantation. However, while preliminary data support these associations, the precise mechanisms and clinical implications remain poorly defined and require further study [22].

Interventions aimed at restoring a healthy gut microbiota, such as the use of probiotics, prebiotics, or dietary modifications, have been proposed as potential adjunct therapies in the management of autoimmune conditions. Still, their application in the context of thyroid autoimmunity and fertility remains investigational.

Although the role of the gut microbiota in modulating immune and reproductive function is an emerging and promising field, its therapeutic application remains largely speculative. Current evidence suggests associations between dysbiosis, systemic inflammation, and autoimmune thyroid disease, but causality and mechanisms remain poorly understood. Clinical trials investigating interventions such as probiotics, prebiotics, or dietary modulation in the context of fertility are scarce, and their efficacy remains unproven [23,34]. While the review acknowledges this uncertainty, the limitations of current microbiota-targeted strategies could be more clearly emphasized. Caution is therefore warranted in translating these early findings into clinical practice without robust randomized controlled trials.

To synthesize the current status of gut microbiota-related interventions in reproductive care, Table 4 summarizes the available evidence and clinical implications.

Table 4 summarizes the current evidence and clinical recommendations regarding microbiota-targeted interventions in the context of female fertility, particularly in patients with Hashimoto’s thyroiditis. It provides a concise overview of four key strategies—probiotics, prebiotics, dietary modifications, and fecal microbiota transplantation (FMT)—each positioned at different stages of scientific validation. Probiotics are the most widely discussed intervention, with limited pilot studies suggesting potential immunomodulatory benefits. However, the absence of robust randomized controlled trials (RCTs) means their use remains investigational and cannot be recommended for routine clinical care in fertility management [22,23,37]. Prebiotics, which aim to enhance the growth of beneficial gut bacteria, have mostly been studied in preclinical or animal models. While conceptually promising, their direct impact on human reproductive outcomes has not been confirmed, and thus they remain outside clinical guidelines [23,34]. Based on observational data, dietary modifications, such as increased fiber intake or anti-inflammatory diets, show the most practical promise. Some studies have linked improved gut health with hormonal balance and reduced systemic inflammation. Still, these findings are correlational and do not confirm causality or direct fertility benefits [22,23]. FMT (Fecal Microbiota Transplantation) is at a purely theoretical stage in reproductive medicine. While it has successfully treated certain gastrointestinal disorders, no clinical evidence supports its application in endocrine or reproductive autoimmune diseases, making it unsuitable for current use [34,38,39,40,41,42]. As such, understanding the gut–thyroid–reproduction axis represents a promising but nascent field of research, offering opportunities for novel therapeutic strategies that go beyond traditional hormonal or immunological treatments.

## 7. Limitations and Future Perspectives

Despite the growing body of literature exploring the link between Hashimoto’s thyroiditis and female fertility, several limitations persist in current research. Firstly, a substantial proportion of the available evidence derives from observational or retrospective studies, which limits the ability to establish causality and weakens the generalizability of the findings [15]. Randomized controlled trials (RCTs) assessing levothyroxine therapy in euthyroid women with thyroid autoimmunity remain scarce, and results from existing trials are often contradictory [16,37].

Secondly, the heterogeneity in defining subclinical hypothyroidism, differences in ART protocols, and lack of standardized inclusion criteria across studies introduce variability that complicates meta-analyses and comparative evaluations [18]. Furthermore, many investigations fail to control for confounding factors such as age, BMI, presence of other autoimmune diseases (e.g., celiac disease, PCOS), or baseline ovarian reserve, which are all known to affect fertility outcomes. Another key limitation involves the emerging area of microbiota research. While preliminary findings suggest an association between gut dysbiosis and thyroid autoimmunity, the underlying mechanisms remain speculative, and no high-quality clinical trials currently validate the use of microbiota-targeted therapies (e.g., probiotics, prebiotics) in this context. In addition, novel single-cell transcriptomic analyses suggest localized immune dysregulation at the endometrial level, further emphasizing the need for mechanistic studies exploring tissue-specific immune environments during the implantation window [24].

One notable limitation across the reviewed studies is the inconsistency in defining subclinical hypothyroidism, with TSH cut-off values ranging from 2.5 to 5.0 mIU/L [13,14,30]. These diagnostic variations can significantly affect the interpretation of ART outcomes. Furthermore, heterogeneity in ART protocols—including differences in ovarian stimulation regimens, fertilization techniques (IVF vs. ICSI), and embryo transfer criteria—introduces additional variability, complicating comparisons between studies and meta-analyses [12,31,33].

Another key limitation across current studies is the lack of control for important patient-related variables, including age, body mass index (BMI), presence of comorbid autoimmune disorders (e.g., celiac disease, PCOS), and baseline ovarian reserve parameters such as anti-Müllerian hormone (AMH) or antral follicle count (AFC). These factors significantly influence reproductive outcomes and should be routinely incorporated into study design and statistical models to enhance interpretability and generalizability [28,33,43,44,45,46].

One of the limitations of this review is its exclusive focus on female reproductive outcomes. Although Hashimoto’s thyroiditis predominantly affects women, emerging evidence suggests that thyroid dysfunction and autoimmune processes may also influence male fertility, particularly through altered hormone levels and impaired spermatogenesis. These aspects were beyond the scope of the present review and warrant future investigation.

To address these gaps, future research should focus on well-designed, large-scale prospective studies that stratify patients by clinical phenotype and explore the efficacy of individualized treatment approaches, including immunomodulation and gut microbiota modulation, as adjuncts in reproductive medicine.

Beyond clinical efficacy, the personalization of fertility treatments in women with autoimmune thyroid disorders raises important ethical considerations, including equitable access to care, informed consent regarding immunomodulatory therapies, and the psychological burden of repeated IVF failure. Patient-centered approaches integrating medical, emotional, and ethical perspectives are essential for optimizing outcomes and ensuring respectful, individualized care [18,47,48,49].

## 8. Conclusions

Thyroid autoimmunity, particularly Hashimoto’s thyroiditis, exerts a multifaceted influence on female fertility. Its impact extends beyond overt thyroid dysfunction, encompassing subclinical hormonal imbalances, autoimmune-mediated ovarian impairment, and altered immune tolerance that may compromise embryo implantation. These effects are mediated through a complex interaction of genetic susceptibility, environmental triggers, and immune dysregulation.

Despite normalization of thyroid hormone levels through levothyroxine therapy, women with thyroid autoimmunity—especially those who are euthyroid—may still face increased risks of infertility, miscarriage, and suboptimal outcomes in assisted reproductive techniques such as IVF and ICSI. This underscores that thyroid antibody positivity should inform fertility evaluations rather than thyroid hormone status alone.

Clinical guidelines increasingly emphasize individualized treatment approaches, recommending close monitoring of TSH levels and consideration of levothyroxine therapy in selected cases, particularly where TSH is elevated or miscarriage risk is high. Moreover, emerging evidence highlights the potential role of gut microbiota in modulating systemic immunity and reproductive success, offering a promising new avenue for therapeutic intervention.

While the review emphasizes the need for personalized management, it offers limited discussion on patient-specific variables that may influence fertility outcomes in Hashimoto’s thyroiditis. Factors such as age, presence of comorbid autoimmune conditions (e.g., PCOS, celiac disease), body mass index (BMI), and variability in response to levothyroxine therapy are all known to affect both natural and assisted conception. For instance, younger patients with isolated anti-TPO positivity may exhibit different reproductive trajectories compared to older women with multiple autoimmune markers and subclinical hypothyroidism. Understanding these inter-individual differences is crucial for tailoring treatment protocols and optimizing outcomes, particularly in the context of assisted reproductive technologies. Future research should therefore stratify findings by demographic and clinical subgroups to refine therapeutic recommendations and enhance clinical decision-making.

Given the heterogeneity of findings across studies and the evolving nature of the evidence base, further high-quality, large-scale research is needed. This should clarify the benefits of tailored thyroid hormone supplementation and explore novel immunomodulatory strategies targeting the gut-thyroid-reproductive axis.

Ultimately, optimizing reproductive outcomes in women with Hashimoto’s thyroiditis requires a multidisciplinary, personalized approach that integrates endocrinology, immunology, and reproductive medicine.

## Figures and Tables

**Table 1:** Immune mechanisms involved in Hashimoto’s thyroiditis and fertility.

Immune Factor	Effect on Fertility	Clinical Evidence
Anti-TPO antibodies	↑ Risk of miscarriage, ↓ implantation rate	Meta-analyses; associated with 2–4× miscarriage risk [3]
Anti-Tg antibodies	↑ Risk of immune ovarian damage	Observed in ovarian dysfunction in autoimmune settings [3,19,20,21]
IL-17α	↓ Oocyte quality and implantation potential	Elevated in Hashimoto patients; correlates with lower pregnancy rates [13,22]
NK cells	↓ Endometrial receptivity, ↑ inflammation	Increased activity in infertile women with TAI [13]
KIR-HLA-C mismatch	↑ Implantation failure risk	Linked with poor ART outcomes in observational studies [13,19,20,21,22,23]

Note: Directional arrows are used to clarify the impact of each immune factor. ↑ Represents an increase in risk or activity (e.g., higher miscarriage rate, increased immune activation), while ↓ signifies a reduction in function (e.g., decreased implantation potential, impaired receptivity).

**Table 2:** Hormonal and ovarian reserve markers in women with Hashimoto’s thyroiditis [9,10,11,25,26,27,28].

Parameter	Normal Role in Fertility	Impact in Hashimoto’s Thyroiditis	Clinical Implication
AMH (Anti-Müllerian Hormone)	Reflects ovarian reserve; lower levels indicate reduced fertility potential	↓ AMH levels observed in subclinical hypothyroidism	May indicate premature ovarian insufficiency in euthyroid women with Hashimoto’s [9,10,11,25]
FSH (Follicle-Stimulating Hormone)	Stimulates follicle development; high levels may indicate ovarian aging	↑ FSH levels are reported due to impaired ovarian feedback	Requires monitoring to guide ART stimulation protocols [9,10,11,25,26]
TSH (Thyroid-Stimulating Hormone)	Regulates thyroid hormone production; optimal range critical for ovulatory function	↑ TSH is standard, even in subclinical cases, and may impair folliculogenesis	TSH normalization is critical before ART initiation [27]
FT4 (Free Thyroxine)	Supports metabolic and reproductive function; needed for endometrial receptivity	FT4 may remain normal, but it fluctuates in some patients with TAI	Needs regular assessment to ensure euthyroidism [28]
AFC (Antral Follicle Count)	Assesses follicle pool via ultrasound; fewer follicles reflect diminished reserve	↓ AFC reported in women with autoimmune thyroid disease	Can help determine candidacy and response to ART [25,28]

Note: The arrows column indicates the direction of physiological change associated with autoimmune thyroid dysfunction. ↑ Denotes an increase, e.g., elevated hormone levels or risk, while ↓ indicates a decrease (e.g., reduced hormone levels or biological function).

**Table 3:** ART outcomes in women with and without thyroid autoimmunity.

ART Procedure	Outcome Without Thyroid Autoimmunity	Outcome with Thyroid Autoimmunity	Observed Effect
IVF [In Vitro Fertilization]	38% clinical pregnancy rate	34% clinical pregnancy rate	↓ implantation and ↑ miscarriage [30]
ICSI [Intracytoplasmic Sperm Injection]	42% clinical pregnancy rate	28% clinical pregnancy rate	↓ fertilization, ↓ implantation, ↑ miscarriage [30]

Note: Arrows indicate the direction of clinical changes associated with thyroid autoimmunity. ↓ Denotes a decrease in outcomes such as fertilization or implantation rates, while ↑ it reflects an increase in adverse consequences, notably miscarriage.

**Table 4:** Current evidence on microbiota-based interventions in fertility.

Intervention Type	Evidence Status	Clinical Recommendation
Probiotics	Limited pilot studies, no RCTs	Investigational only [22,23,34]
Prebiotics	Experimental models	Not recommended for clinical use [23,34]
Dietary modifications	Observational evidence	Potentially beneficial, not proven [22,23]
Fecal microbiota transplant (FMT)	Theoretical only	Not applicable in current practice [34]
